# Editorial: Morphogenic Cascades Underlying Regeneration and Plasticity After Nervous System Injury

**DOI:** 10.3389/fcell.2021.753777

**Published:** 2021-08-30

**Authors:** David Lutz, Monika von Düring, Irina I. Stoyanova

**Affiliations:** ^1^Department of Neuroanatomy and Molecular Brain Research, Ruhr University Bochum, Bochum, Germany; ^2^Department of Anatomy and Cell Biology, Medical University Varna, Varna, Bulgaria

**Keywords:** extracellular matrix, nervous system injury, adhesion molecules, ghrelin, trauma

Injury of the nervous system entails a broad spectrum of pathological events with different etiology and clinical manifestation. Better understanding of these processes could help prevent propagation of the degeneration into the tissue surrounding the injury and stimulate repair. However, the scenario of nervous system regeneration is complex and requires activation of a cohort of cellular and extracellular cascades by molecules that play essential roles in nervous system development, e.g., by initiating neurogenesis, reorganization of synaptic contacts and remodeling of the extracellular matrix. This Research Topic includes studies on *in vitro* and *in vivo* models of injuries of different parts of the nervous system yielding mechanistic insights into the roles of developmentally related molecules in regeneration upon brain trauma, heat and ischemic stroke, and inflammatory conditions accompanying neurodegeneration.

Paramount importance is laid on the understanding of the highly complex microenvironment within the core of and around the injury site, thus following the ultimate aim of design of novel therapeutic strategies centered on molecules which target specific pathways beneficial for regeneration. These are exemplified by studies of extracellular matrix components such as tenascin (TnC), insulin-like growth factor-1 (IGF-1), and the metalloproteinase ADAM10, all of which modulate signaling cascades underlying nervous system repair, in particular by stimulating neuronal plasticity and other morphogenic events required for regeneration after injury.

TnC has a key role in intercellular communication providing fine tuning of the balance between excitation and inhibition as well as in immunomodulation. The molecule orchestrates neurogenesis at early developmental and late postnatal stages (Tucić et al.). Similarly to TnC, IGF-1 also increases neurogenesis after traumatic brain injury (TBI) and improves the long-term survival rate of the hippocampal newborn neurons by sustaining their functionality. As a potential mechanism for these effects the authors have considered the activation of the mammalian target of rapamycin (mTOR), which regulates proliferation, axonal and dendritic growth. Moreover, controlled cortical impact TBI triggers mTOR activation in the neurogenic niches in a time-, region-, and injury severity-dependent manner, and the effect is amplified with conditional overexpression of IGF-1. As shown by Littlejohn et al., IGF-1 overexpressing mice exhibit upregulated posttraumatic neurogenesis, with a higher density of post-trauma-born granule cell layer neurons at 10 days after injury. Intriguingly, IGF-1 does not affect delayed astrocytic activation of mTOR in the dentate gyrus.

Unlike the other two components of the extracellular matrix, ADAM10 is understood by Appel et al. to exert a negative effect on recovery of injured brain tissue. This metalloproteinase can cleave almost 100 different substrates and dysregulation of its proteolytic activity is linked to various types of cancer, immunity and inflammatory pathologies. The authors have demonstrated that inhibition of the metalloproteinase ADAM10 has a beneficial effect on the recovery of the nervous system after TBI characterized by reduced brain tissue loss, axonal injury and pro-inflammatory gene expression. Furthermore, inhibition of ADAM10 attenuates expression of metalloproteinase genes associated with neuronal cell death after TBI and maintenance of the blood-brain-barrier, yet not of blood-brain-barrier's overall integrity. ADAM10-inhibition also reduces axonal injury and alters gene expression of glutamate receptor subunits.

Another highlight of the current Research Topic is the work of Chongtham et al. that is focused on the analysis of the transcriptomic response of the microenvironment to brain injury, in search of novel markers for precision in diagnostic of complex pathologies. In their model of transient global cerebral ischemia in the primate subventricular zone (SVZ) the authors have identified 541 up-regulated and 488 down-regulated genes (see also www.monkey-niche.org). The up-regulated genes revealed a profile which is typically displayed by quiescent stem cells and astrocytes, and the majority of these genes is mapped to the subependymal layers on the striatal or callosal aspects of the SVZ. Of note, the substantial number of up-regulated genes in the ependymal layer suggests implication of ependyma into stem cell biology. Based on the transcriptome analysis, the authors have identified several novel markers for primate SVZ including the apelin receptor, which is strongly expressed upon ischemia.

Further important observations on organotypic hippocampal slices made by Weninger et al. have suggested that the heat shock, which causes granule cell dispersion in patients with temporal lobe epilepsy, when applied to the slices, does not affect the reelin-expressing Cajal-Retzius cells in the dentate gyrus but rather evokes a massive microgliosis. These findings underpin microgliosis as a novel marker to distinguish pathological granule cell dispersion from normal morphological variation in pediatric patients with history of febrile seizures without suffering epilepsy.

Our Research Topic suggests that counteracting the complexity of nervous system injury needs either a molecule with pleiotropic functions or cocktails of bio components with specific roles in stimulating or inhibiting the multitude of signaling pathways modulating regeneration. To find an omnipotent therapeutic molecule, however, seems to be quite ambitious a task, giving the complex demands of the regenerating nervous system.

Among the numerous candidates examined so far, the neuropeptide and hormone ghrelin displays very promising therapeutic potential with its ability to cross the blood-brain-barrier, to balance metabolic processes, and to stimulate neuroregeneration and neuronal activity (as reviewed by Stoyanova and Lutz). Ghrelin exercises its cytoprotective and repair capacity through increasing autophagy, beta-oxidation, cell viability and through reducing apoptosis. It activates cell cycle and facilitates a variety of morphogenic and dynamic events such as neurite outgrowth, myelination, synaptogenesis, cell migration and extracellular matrix reorganization. Unclear is still how ghrelin could trigger all these events and the authors suggest that since recovery recapitulates ontogenesis, where ghrelin plays a significant role, one could conclude that the neuropeptide would stimulate developmentally related molecules, neurotrophic and transcription factors if applied as a therapeutic agent.

In conclusion, the Research Topic articles illustrate the complexity of the pathological microenvironment in the injured nervous system and its numerous players, ranging from neural components such as neurons and glial cells with their transmitters, modulators and receptors, the components of the extracellular matrix for vascularization and/or neovascularization, up to the microglia, macrophages and other cells of the immune system with their chemokines and cytokines. These players are equipollent in their contribution to the regeneration of the injured nervous system and demand complex multi-targeting therapeutic strategies which are understood to be much more etiology-oriented rather than symptoms-based.

## Author Contributions

All authors listed have made a substantial, direct and intellectual contribution to the work, and approved it for publication.

## Conflict of Interest

The authors declare that the research was conducted in the absence of any commercial or financial relationships that could be construed as a potential conflict of interest.

## Publisher's Note

All claims expressed in this article are solely those of the authors and do not necessarily represent those of their affiliated organizations, or those of the publisher, the editors and the reviewers. Any product that may be evaluated in this article, or claim that may be made by its manufacturer, is not guaranteed or endorsed by the publisher.

